# Status and prospects of seasonal malaria chemoprevention among children in Sahelian countries: A systematic review and meta-analysis

**DOI:** 10.1371/journal.pgph.0005124

**Published:** 2025-09-12

**Authors:** Medard Djedanem, Noura Mamane Salé, Elhadji Yacoudima Yacoubou Mahaman Aminou, Jean Testa, Ronan Jambou

**Affiliations:** 1 CERMES, Niamey, Niger; 2 EPICENTRE, Niamey, Niger; 3 Université Nazi Boni, Bobo-Dioulasso, Burkina Faso; 4 Service de Santé de la Garde Nationale du Niger, Niamey, Niger; 5 Laboratoire RETINE, Faculté de médecine de Nice, Nice France; 6 Institut Pasteur, Global Health Department, Paris, France; PLOS: Public Library of Science, UNITED STATES OF AMERICA

## Abstract

In areas with seasonal malaria transmission, seasonal malaria chemoprevention (SMC) involves giving children a three-day course of sulfadoxine-pyrimethamine and amodiaquine once a month during the transmission season. This strategy has been used for ten years for children under five years of age. The logistical cost is affordable in malaria-endemic countries and varies slightly depending on accessibility. This systematic review and meta-analysis aims to better assess the effectiveness of seasonal malaria chemoprevention in reducing malaria incidence and prevalence in Sahelian countries, ten years after its introduction. This review followed the Preferred Reporting Items for Systematic Reviews (PRISMA) 2020 guidelines, and was based on Google scholar, MEDLINE (PubMed), the Cochrane library, African Journal Online, and Index Medicus African to compile its data. The combination of keywords such as “malaria”, “*Plasmodium*”, “malaria chemoprevention”, “Sahel”, “Efficacy”, “Resistance”, as well as Boolean operators (AND, OR) were used to inventory studies published between 2013 and 2023. Eligible studies included randomized clinical trials (RCTs), non-randomized trials, prospective cohort studies, intervention studies and observational studies. For randomized trials (RCTs), Cochrane’s Risk of Bias 2 (RoB 2) tool was used to specifically assess the risk of bias in randomized controlled trials. And for non-randomized trials and observational studies, we applied the ROBINS-I (Risk of Bias in Non-randomized Studies of Interventions) tool, which included a meta-analysis of the studies. The study protocol was registered under the number (PROSPERO registry CRD42023413920), and R software was used for the meta-analysis. The meta-analysis shows that SMC is effective in reducing the incidence of uncomplicated malaria, severe malaria and mortality in children under five. Compared with control groups a reduction in the burden of malaria was observed in children receiving SMC. SMC appears to effectively reduce the incidence of malaria in children under five. However, it should be noted that SMC is always used alongside other prevention strategies, such as indoor residual spraying and long-lasting insecticide-treated nets. However, the epidemiological context of the Sahelian region is changing, and the strategy must be adapted to address the persistence of transmission during the dry season and the increase in malaria cases among older children.

## Introduction

Sub-Saharan Africa remains the world’s main malaria transmission zone and still experiences high morbidity and mortality rates. Despite low rainfall, Sahelian West Africa is also heavily impacted, but transmission in this area is seasonal. Arid countries such as Niger and Burkina Faso are among the top six countries in Africa with the highest rates of malaria transmission, and children under five are the most at risk [1]. Ten years ago, alongside other strategies such as insecticide-treated bed nets, Seasonal Malaria Chemoprevention (SMC) was introduced to areas with seasonal malaria transmission. SMC was formerly known as Intermittent Preventive Treatment (IPT). This treatment aims to maintain therapeutic concentrations of antimalarial drugs in the blood during the period when the risk of contracting malaria is highest. [2]. Children without malaria receive a complete, systematic antimalarial treatment based on sulfadoxine-pyrimethamine and amodiaquine (SPAQ) every month for three to four days. Children aged 3–11 months receive a single dose of 250/12.5 mg of SP and three doses of 57.5 mg of AQ spread over three days. Children aged 12–59 months receive 500/25 mg of SP in a single dose and 153 mg of AQ in three doses. AQ is rapidly metabolized in the body to desethylamodiaquine (DEAQ) [3]. Thanks to the long half-life of both molecules, the treatment remains active for around 28 days after administration [4,5]. Treatment is repeated monthly for three to five months during the rainy season [6]. This SMC is not administered to acutely ill children, children with HIV, or those with allergies to any of the drugs used [7]. Over the last ten years, SMC has been deployed, particularly in Sahelian regions. Its effectiveness is now well-demonstrated, although it should be noted that SMC is always combined with vector control strategies, such as using long-lasting insecticide-treated nets (LLINs), residual insecticide spraying, and destroying breeding sites. Several African countries have reported that this strategy is highly effective. They have seen a 55% to 73% reduction in the incidence of uncomplicated malaria in children under five, a 26% reduction in the incidence of severe malaria, and a 42% to 48% reduction in malaria mortality [8–10]. However, it remains difficult to attribute these successes solely to SMC. What is the ten-year assessment of this strategy in practice? Was it easy to implement? Most importantly, has it been effective in protecting children?

This evaluation is urgent, as ecosystems in Africa—particularly those in the Sahel—are rapidly evolving due to urbanization and climate change [11–16], impacting malaria transmission. For instance, increased rainfall creates residual pools, which allow transmission to extend from December to February or March in Sahelian countries [17–22]. Should additional treatment cycles be considered during the dry season? Should this strategy be implemented in Sahelian regions that receive less than 300 mm of rainfall per year? Should other age groups be included in the strategy, and what would be the cost?

These questions must be answered to develop a strategy for the future, which requires us to review the last ten years of implementation.

This systematic review and meta-analysis, based on published studies, provides elements to evaluate the effectiveness of SMC in reducing the incidence, prevalence, and case-fatality rate of malaria in children under five in Sahelian countries. The review also highlights the difficulties encountered and the prospects for this strategy.

## Materials and methods

### Study protocol

The inclusion/exclusion strategy for publications used in this study is based on the PRISMA (Preferred Reporting Items for Systematic Reviews and Meta-Analyses) method [23]. The protocol has been registered with the International Prospective Register of Systematic Reviews (PROSPERO) under the number CRD42023413920 [16].

#### Research strategy.

Studies were selected according to the PRISMA checklist, and the PICOS (Population Intervention Comparison Outcome Study design) criteria [23]. Several search engines, including “Google scholar, MEDLINE(PubMed), the Cochrane Library, African Journal Online, and Index Medicus African”, were used to search for studies published within the last ten years (January 2013 to December 2023). The year 2013 was chosen because SMC was introduced in most Sahelian countries in 2012 [24–27]. All articles published up to December 2023 were reviewed focusing on the efficacy of reducing malaria incidence, prevalence, and mortality. The search was based on a combination of keywords and Medical Subject Headings (MeSH), e.g., “malaria chemoprevention”, “Sahel”, “plasmodium”. “Malaria chemoprevention”, “Sahel”, and “plasmodium”, the search was then secondarily refined with the keywords, “efficacy”, “impact”, “global coverage”, “Burkina Faso”, “Mali”, “Niger”, “Chad”, “Senegal”, “Gambia”, “Cameroon”, “Nigeria”, “Guinea”, and “Mauritania”, as well as the relevant Boolean operators (AND, OR). Keywords were applied to the title, abstract and text.

#### Selection of studies.

The studies included in the meta-analysis met the following predefined criteria: study design, age of participants, intervention, and outcome measures. The literature search included studies published between 2013 and 2023. Eligible studies were randomized and non-randomized clinical trials, prospective studies, observational studies, and intervention studies that reported the efficacy of SMC in Sahelian countries. Animal and in vitro studies were excluded. Two people independently selected studies based on titles, abstracts, and full texts via the Rayyan platform [28,29]. Neither researcher could consult the other’s activity. If the two researchers disagreed about including an article, a third researcher was consulted to make the final decision. If the abstracts were not explicit, the full-text articles were downloaded for review. Zotero bibliographic software was used to manage, store, and organize references. The primary objective of this review was to evaluate the effectiveness of SMC in preventing malaria in children under five years old in the Sahel region.

#### Data extraction.

Studies were selected based on their titles, contents, and abstracts. Data were extracted using a registration form that included the following: (i) the title of the article or journal, the authors’ names, the country in which the study was conducted, the languages used, and the years of publication; (ii) the methodological features, such as the study design, the sample size, the gender of the participants, the full details of the intervention, and the statistical analyses; (iii) the main results, such as the reduction in incidence and prevalence; and (iv) the conclusions. For non-randomized trials and observational studies, we applied the ROBINS-I (Risk of Bias in Non-randomized Studies of Interventions) tool. Randomized trials, intervention studies, and biases were assessed via the revised ROBINS-I tool (Table 3). For randomized trials (RCTs), the Cochrane Risk of Bias 2 (RoB 2) tool was used to assess the risk of bias in RCTs specifically (Table 4).

We excluded studies based on the following criteria: language of publication (other than English or French), duplicates, unavailable abstracts or full text, inappropriate design, or study population. All non-primary literature, retrospective studies, case reports, and in vitro experiments were excluded.

### Meta-analysis process

#### Data selection.

A standardized data extraction form was used to extract data from the included studies (Tables 1 and 2). Key variables included study characteristics, participant demographics, intervention details, and outcome measures. Two independent members of the review team assessed the studies’ methodology using quality assessment forms according to the criteria of the Cochrane Handbook for Systematic Reviews of Interventions [40,41]. For observational studies, the Newcastle-Ottawa Scale (NOS) was used [42]: (i) the NOS assesses study quality in three areas: (ii) study selection (representativeness, exposure assessment, and control selection); (iii) comparability (adjustment for major confounders); and (iv) outcomes (measuring outcomes against self-report) [43]. The risk related to each included study was presented using the ROBINS-I process (https://methods.cochrane.org/robins-i) and RoB 2.

**Table 1:** Full search strategies for all databases, registers and websites.

Data base	Search terms	Search result
Pub Med	((((((((((((((effect[MeSH Terms]) OR (effectiveness[MeSH Terms])) OR (impact[MeSH Terms])) OR (evaluation[MeSH Terms])) OR (benefit[MeSH Terms])) OR (seasonal malaria chemoprevention[MeSH Terms])) OR (intermittent preventive treatment[MeSH Terms])) OR (IPTi[MeSH Terms])) OR (IPTc[MeSH Terms])) OR (intermittent preventive therapy[MeSH Terms])) AND (malaria[MeSH Terms])) OR (chemoprophylaxis[MeSH Terms])) AND (malaria[MeSH Term) AND (children) AND (sahel)	71
Google scholar	FIRST STEP (“efficacy”, “effect”, “impact” AND “seasonnal malaria chemoprophylaxy” AND “Sahel”) SECOND STEP “Plasmodium falciparum” AND “ falciparum malaria” AND “IPTi” OR “IPTc” AND “sahel”	473
Cochrane library	(“efficacy” OR “impact” OR “full program”) AND (“seasonnal malaria chemoprophylaxy” AND “sahel” AND “malaria”)	178
African joutnal online	(“efficacy” OR “impact” OR “full program”) AND (“seasonnal malaria chemoprophylaxy” AND “sahel” AND “malaria”)	71
Index Medicus African	(“efficacy” OR “impact” OR “full program”) AND (“seasonnal malaria chemoprophylaxy” AND “sahel” AND “malaria”)	4

**Table 2:** Strategies PICOS. PICOS strategies and eligibility.

PICOS strategies	Integration criteria	Exclusion criteria
P: Population	Children under five years of age, of both sexes, who reside in a Sahelian country and have received SMC to prevent malaria during periods of high transmission..	
I: Intervention	Studies have used oral combinations of SPAQ*. Participants must have received at least one dose of SMC during the four-month distribution period.	
C: Comparaison	Comparisons of standard treatments	Animal and in vitro studies
O: Outcome	The primary outcome is a reduction in the incidence and prevalence of malaria. The secondary outcome is the occurrence of adverse events following SMC administration in children under five.	Studies that do not report on the effectiveness of SMC in Sahelian countries
S: Study design	Both randomized and non-randomized clinical trials, as well as prospective cohort and intervention studies, have reported on the therapeutic efficacy of SMCs in preventing malaria in Sahelian countries.	animal experiments, retrospective studies, non-primary literature, and in vitro studies

* suphadoxine-pyrimethamine-amodiaquine.

#### Data synthesis strategy.

A meta-analysis was conducted to estimate the overall effect of SMC in Sahelian countries. A random-effects model was employed to account for heterogeneity among the studies. Heterogeneity was assessed using the I² statistic, which measures the percentage of total variation between studies. Substantial heterogeneity was defined as a value of 50% or more. Continuous variables were compared using standardized mean differences, dichotomous outcomes using odds ratios (OR). A sensitivity analysis was conducted to evaluate the robustness of the results. Studies with a high risk of bias were excluded in order to assess the impact of study quality on the results. Subgroup analyses were performed to explore the effects of different intervention types, dosages and intervention durations. Publication bias was explored using funnel plots. However, due to the small number of studies included (less than 10), interpretation remains limited. The Egger’s test was not applied. A complementary search was carried out in clinical trial registries such as Clinical Trials.gov (National Institutes of Health) and the WHO ICTRP (World Health Organization - International Clinical Trials Registry Platform).

The statistical software R version 4.3.1 was used to implement a random-effects meta-analysis model according to Der Simonian and Laird. Statistical heterogeneity was graphically analyzed via a Forest plot and tested using Cochran’s chi-squared (χ2) and I^2^ tests to assess the significance of heterogeneity [44]. Thresholds were also defined for the studies included in the meta-analysis to determine heterogeneity: I² < 0.25 = low heterogeneity, I² between 0.25 and 0.5 = moderate heterogeneity, and I² > 0.5 = high heterogeneity. Adjusted malaria prevalence was calculated using a hierarchical model of multivariate meta-analysis to account for variance due to study design and country [45,46]. Funnel plot inspection and Egger’s test (p < 0.10) were used to detect publication bias. A p < 0.05 value was considered significant [47]. The outcomes of interest included malaria incidence, adverse events, and adherence to SMC regimens. Data on the targeted population, intervention duration, and study location were also collected.

#### Key factors influencing the effectiveness of SMC.

The analysis highlights four major determinants of the efficacy of seasonal malaria chemoprevention (SMC): (i) coverage, which is difficult to achieve in rural areas and is therefore often below the target of 80%; (ii) adherence, which sometimes declines after the first dose and is correlated with level of education and distance from health centers; (iii) drug resistance, which is influenced by dhfr/dhps mutations that reduce the efficacy of sulfadoxine-pyrimethamine in areas where SMC is implemented; and (iv) the climatic context, in which the length of the malaria season and rainfall influence the timing of administration. These interdependent factors underscore the necessity of contextualized interventions to optimize the impact of SMC [1,7,17].

## Results

### Results of the literature search

**Fig 1:**
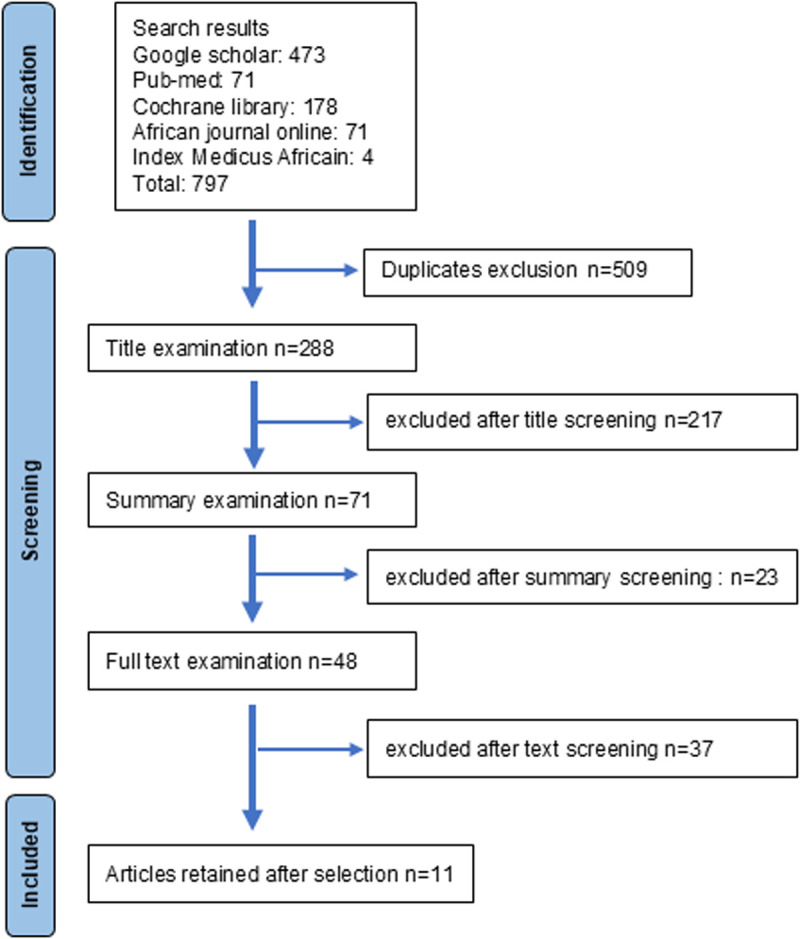
Selection of manuscripts for the review. This process adheres to the PRISMA recommendations.

The goal of this selection was to compile a set of studies that provide reliable evaluations of SMC (see Tables 1 and 2). Many publications report on other topics but only cite SMC in passing. Out of a compilation of 797 studies, 509 duplicates were excluded. Of the remaining 288 articles, 217 were excluded after a title review, 23 after an abstract review, and 37 after a full-text review. In the end, 11 articles were included in this systematic review and meta-analysis (Fig 1, S1 Table).

#### Characteristics of included studies.

A summary of the included studies is presented in S2 Table. A total of 11,232,505 participants were included in the eleven selected studies (Table 3). Seven of the studies were observational and included data on treatment efficacy, the number of monthly treatment cycles over the course of a season, costs, logistics, and the impact on malaria incidence in non-malarial children under five and on allergies in Sahelian countries that have implemented this strategy since the first WHO recommendations. Two of the studies were clinical trials, one was a prospective cohort study, and one was a descriptive cross-sectional evaluation study. These studies were conducted in several Sahelian countries. Participants ranged in age from 3 to 59 months, and most studies did not specify the proportion of boys to girls.

**Table 3:** Characteristics of studies included in the meta-analysis (efficacy/impact).

Journal	Authors (ref)	Years of publication	Type of design	Sample size coated	Age range included in months	Study duration in months	Adverse effects	Effectiveness %
Journal of Infectious Diseases 221(1): 138–145.	Attaher, et al. (39)	2020	Clinial trial	50	12-59	5	NS*	63
The Lancet 396(10265): 1829–1840.	Baba, et al. (40)	2020	Observational study	11 201 946	3-59	24	36	88,2
PLoS Medicine 17(8): e1003214.	Cairns, et al. (41)	2020	Random clinical trial	19570	3-59	36	NS*	87
PLoS Medicine 13(11): e1002175.	Cisse, et al. (42)	2016	Prospective observational	NP	3 - 59	36	NS*	69
Malaria Journal 16(1): 325.	Diawara, et al. (43)	2017	Observational study	NP	3-59	5	NS*	NS*
Am J Trop Med Hyg 101(5 Supplement): 314.	Diawara, et al. (44)	2019	Observational study	651	3-59	4	0,11	81
Malaria Journal 19(1): 103.	Issiaka, et al. (45)	2020	Observational study	6638	3-59	1	NS*	80,4
Malaria Journal 19(1): 137.	Konate, et al. (46)	2020	Cohort follow-up		3-59	NS*	NS*	77
Int J Env Res & Public Health 17(18): 11.	Maiga, et al. (47)	2020	Observational study	1332	3-59	36	NS*	87,6
Am J Trop Med Hyg 97;5 Suppl: 134.	Manga, et al. (48)	2017	Observational study	2008	3-59	1	NS*	NS*
Health Sci and dis	Oumar A A, et al. (49)	2021	Cross-sectional study	310	3-59	4	0,69	56

* NS: NOT SPECIFIED.

During these studies, the first dose of AQSP was usually administered under the supervision of a health worker and/or community relay, and doses on days two and three were given to parents for administration at home. The results showed an overall 75.2% reduction in the incidence of uncomplicated malaria, a 26% reduction in the incidence of severe malaria, and a 48% reduction in malaria mortality.

#### Selection of studies for meta-analysis.

Eleven studies were included in the meta-analysis using both a common-effect and a random-effects model, and bias was analyzed (Table 4). In the common-effects model, the overall odds ratio (OR) for these studies (Table 5) was 1.98 (95% CI [1.89, 2.07]), whereas in the random-effects model, the OR was slightly lower at 1.63 (95% CI [1.33, 1.94]). Z-tests for both models showed significant values (43.51 for the common-effects model and 10.53 for the random-effects model; p < 0.0001). Studies with fairly homogeneous results were selected for meta-analysis together.

**Table 4:** Reasons for risk of bias (ROBINS-I).

study (ref N°)	study design	Confounding	Selection participants	Classification of interventions	Deviation from intended interventions	Missing Outcome data	Outcome measurements	Selcetion of result reported	Overrall RoB
Attaher, et al. [30]	clinical trial	Serious	Low	Moderate	Moderate	Critical	Low	Low	Serious
Baba, et al. [31]	observational study	Critical	Low	Moderate	Moderate	Critical	Low	Low	Critical
Cisse, et al. [32]	prospective observational	Critical	Low	Moderate	Moderate	Critical	Low	Low	Critical
Diawara, et al. [33]	observational study	Critical	Low	Moderate	Moderate	Critical	Low	Low	Critical
Diawara, et al. [34]	observational study	Serious	Low	Moderate	Moderate	Critical	Low	Low	Critical
Issiaka, et al. [35]	observational study	Serious	Low	Moderate	Moderate	Critical	Low	Low	Serious
Konate, et al. [36]	cohort study	Serious	Low	Moderate	Moderate	Critical	Low	Low	Serious
Maiga, et al. [37]	observational study	Serious	Low	Moderate	Moderate	Critical	Low	Low	Serious
Manga, et al. [38]	observational study	Serious	Low	Moderate	Moderate	Critical	Low	Low	Serious
Aboubacar, et al. [39]	transversal study	Critical	Low	Moderate	Moderate	Critical	Low	Low	Critical

**Table 5:** Meta-analysis statistics.

Estimated combined effect (log-odds)	Estimated combined effect (OR)	Global effect (IC 95%)	heterogeneity I² (%)	H² (total heterogeneity/ sampling variability)	tau² (variability between studies)	tau τ² (root of tau²)	p-value for overall effect					Conclusions keys
0,38	1,46	[1.37, 1.57]	27,38	1,38	0,0017	0,0412	0,0001					
**Studies**	**Years of** **Publication**	**study population**	**Effect main (RR/OR)**	**Reduction** **mortality**	**Reducing hospitalizations**	**TE (Estimated effect)**	**SE(TE) (Standard error)**	**95% CI (Common model)**	**95% CI (Random Model)**	**Weight** **(Common model) %**	**Weight** **(Random model) %**	
Baba et al.	2020	11 201 946	88.2% (95% CI: 78.7-93.4) over 28 days	Burkina Faso: 42,4%, Gambie: 56,6%	Not reported	2.0721	0.0500	[1.97, 2.17]	_	83.1	24	Reduction in deaths during high transmission, varies by country
Cissé et al.	2016	Children under 10 in Senegal	RR: 0.60 (95% CI: 54–64%) for incidence, p < 0.001	(RR: 0.90, 95% CI: 0.68-1.2)	45% pour formes graves (p = 0,031)	1.0865	0.3000	[0.50, 1.67]	_	2.3	12.9	Coverage >80%, marked reduction in confirmed clinical cases
Diawara et al.	2017	633 (Kita 310 & 323 Bafoulabé)	reduction 40% of prevalence of parasitaemia (OR= 0,60; IC 95%: 0,41–0,89)	Not reported	Not reported	1.8560	0.2000	[1.46, 2.25]	_	5.2	17.6	Implementation of SMC resulted in a significant 40% reduction of parasitaemia in the extended age group, with good coverage 81,2%
Diawara et al.	2019	Children receiving SMC	reduction 23,4% to 18% in the district with intervention and 46% increase in the district without intervention (DD OR= 0,20; IC 95%: 0,04–0,94), P = 0.34 prevalence of malaria illness (2.4 vs 1.9%, P = 0.75)	Not reported	Not reported	1.7233	0.4000	[0.94, 2.51]	_	1.3	9.4	Positive impact on parasitaemia and moderate anaemia
Issiaka et al.	2020	6638	RR: 0,44 (IC 95%: 0,22–0,91), p = 0,027	56% (RR: 0,44, IC 95%: 0,22–0,91)	39% (IRR: 0,61, IC 95%: 0,44–0,84), p = 0,003	1.5280	0.2000	[1.14, 1.92]	_	5.2	17.6	Significant effect on admissions and mortality
Maiga et al.	2020	1332	IR: 0.01 (95% CI: 0.0001-0.09) for infection	Not reported	Not reported	1.1515	0.6000	[0.02, 2.33]	_	0.6	5.3	Marked reduction in malaria incidence between 2012 and 2014
Manga et al.	2017	2008	Parasite prevalence before/after in 2013: 11.8% → 6.1%	Not reported	Not reported	1.3529	0.3000	[0.76, 1.94]	_	2.3	12.9	in 2013, a significant reduction reduction in the prevalence of plasmodium was observed after SMC from 11,8% to 6,1%. In 2015, an opposite trend was noted, with an increase in prevalence from 4,9% before the Campaign to 15,3% after

### Ten years of SMC in a nutshell

#### Which strategy is used?

Since 2012, countries have adopted a fairly uniform strategy based on three doses of AQ and one dose of SP administered over three days. Following WHO recommendations, the treatment is administered every 28 days from the start of the rainy season until the dry season begins (i.e., three to five months). Two countries, Uganda and Mozambique, have more recently begun to implement the strategy [48]. SMC is currently administered in most African and Sahelian countries only to children under five years old [17].

To ensure the 28-day interval between campaigns, most countries use teams that go door-to-door in villages, which incurs significant logistical costs. In some countries, children must attend health centers monthly for treatment, which, in rural areas, poses a significant logistical challenge for families. In order to organize the delivery, teams need an up-to-date census of the population, which is rarely available. In all countries, the second and third doses of AQA are given to parents or guardians for self-administration to their children. In most cases, pharmacovigilance monitoring is inadequate and must be improved to detect adverse effects [48].

#### How much does SMC cost?

The cost of the strategy varies from one country to another depending on the logistics of reaching children in remote areas (including areas of insecurity) and the daily per diem of the agents responsible for distribution. Between 2015 and 2016, ACCESS-SMC conducted a study in seven Sahelian countries to determine the cost and effectiveness of the strategy per child and per delivery. The study revealed a weighted monthly cost of $3.63 per child per year [30]. In Mali, the cost per child was $6.38 for four cycles and $4.69 for three cycles [31]. In Gambia, village health workers administer doses, and the annual cost of SMC per child is 1.63 USD. In Senegal, SMC was administered by health posts. The average cost for 46 posts was estimated at 0.5 USD per child per month (1.50 USD per child per year) [48,49]. SMC is primarily financed by technical and financial partners in Sahelian countries, but ultimately, governments will need to cover the program’s costs.

#### Logistical difficulties.

Thirteen African and Sahelian countries (Benin, Burkina Faso, Cameroon, Chad, Gambia, Ghana, Guinea, Guinea-Bissau, Mali, Niger, Nigeria, Senegal, and Togo) have implemented SMC in their national policies over the past ten years to protect children under five [48]. By 2021, 45 million children under the age of five had benefited from SMC. The population widely accepts this strategy, and children tolerate it well [32]. Covering the target population remains a real challenge in some areas, leading to administrative coverage rates over 100% in some localities, especially when field teams are effective. Another coverage problem is noncompliance with the second and third doses. Mothers and/or babysitters prefer to save these doses to administer to their children when they have a fever. In summary, the main difficulties in deploying SMC are: i) the logistics of reaching hard-to-reach areas and zones of insecurity, ii) the difficulty of finding the same child three to five times during the season, and iii) the difficulty of establishing a census of the target population eligible for SMC [48].

#### Overall results of SMC effectiveness (meta-analysis).

Although it is combined with other strategies, such as the use of long-acting impregnated mosquito nets and indoor residual spraying, the efficacy of seasonal malaria chemoprevention (SMC) in reducing the incidence of uncomplicated malaria is proven in several countries where malaria is endemic but seasonal. These countries claim to have very good administrative coverage rates, often in excess of 100%, which underscores the difficulties associated with estimating target populations in countries that implement SMC.

A study conducted in seven Sahelian countries revealed an overall administrative coverage rate of 53% for a four-passage cycle [11]. However, countries used to report much better results. For example, in 2015, Burkina Faso reported a monthly SMC coverage rate of 92.2%, Chad reported 68.3%, Gambia reported 81.8%, Guinea reported 78.8%, Mali reported 68.3%, Niger reported 61.8%, and Nigeria reported 83%. In 2016, the reported monthly MOH coverage was 96.4% in Burkina Faso; 53% in Chad; 67.4% in Gambia; 86.4% in Guinea; 77.9% in Mali; 75.6% in Niger; and 74.8% in Nigeria [33,34]. By October 2020, Niger had administered SMC to 4,494,046 children between 3 and 59 months of age [35]. In its determination to prevent malaria in children under five, Niger achieved 99.94% SMC coverage by 2021. However, administrative coverage varied by region, ranging from 92.35% in Maradi to 108.38% in Niamey. Similarly [36], Senegal reported administrative coverage of 90%, while Gambia reported 74% [37]. Overall, SMC programs had protected 15.7 million children in 12 Sahelian countries (Burkina Faso, Cameroon, Chad, Gambia, Ghana, Guinea, Guinea-Bissau, Mali, Niger, Nigeria, and Senegal) [38].

Various African countries where malaria is endemic but seasonal have reported reductions in the incidence of malaria. These reductions range from 55% to 73% for uncomplicated malaria, 26% for severe malaria, and 42% to 48% for malaria mortality [17]. A 2020 study in Mali Dangassa health district [39] showed that SMC implementation led to a significant reduction in clinical malaria incidence, observed in 2015 (HR = 0.27, 95% CI: 0.18–0.40) and 2016 (HR = 0.23, 95% CI: 0.15–0.35). Another study [50] found that implementing SMC led to a significant reduction in the incidence of uncomplicated malaria during the transmission season (rate ratio: 0.27, 95% CI: 0.25–0.29 in children under five). However, a more detailed analysis of these studies, set out below, helps to better understand these results.

**Fig 2:**
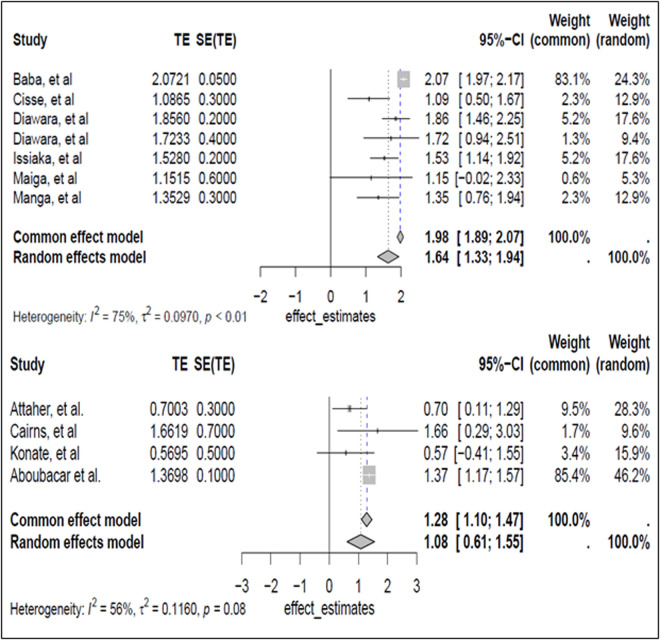
Meta-analysis of the data from the selected studies.

Initially, seven observational study articles with the same design and fairly homogeneous results were pooled to determine the efficacy of AQSP-based treatment in reducing malaria-related morbidity and mortality in children. In these studies, the control group received no treatment. The results of the meta-analysis show a significant difference between the AQSP-treated group and the control group (Fig 2). The pooled effect size was 0.41 (95% CI [0.28, 0.53]), and the z-test statistic was 6.44 (p < 0.01), indicating a significant difference between the treated and control groups. The random effects model yielded the same effect estimate of 0.41 (same CI 95%). Heterogeneity between studies was assessed using Tau², which was zero (indicating no heterogeneity between studies), and I² was also set to zero. These seven studies support a significant reduction in malaria incidence in children under five by SMC. A study by Baba et al. [33] found that SMC was associated with an 88.2% reduction in confirmed malaria cases (CI95% [78.7-93.4]) compared with controls. Additionally, a significant reduction in malaria-related hospital deaths was observed in several countries during periods of high transmission. Cissé et al. [51] reported a 60% (95% CI [54%-64%]) reduction in malaria cases confirmed by rapid diagnostic tests (RDTs) and a 69% (95% CI [65%-72%]) reduction in severe malaria cases in children under five in areas where SMC was implemented. Diawara et al. [52] also observed a significant reduction in malaria and moderate anemia prevalence in the intervention area compared to the control area. The prevalence of parasitemia decreased in the intervention district from 23.4% to 18%. In contrast, it increased in the control district from 29.5% to 46%. Similarly, Issiaka et al. [53] reported that the all-cause mortality rate per 1,000 person-years was 8.29% in control areas without SMC compared to 3.63% in intervention zones with SMC. After adjusting for age and sex, the mortality rate ratio was 0.44 (95% CI [0.22-0.91], p = 0.027), indicating that the mortality rate was 0.44 times lower in intervention zones than in control zones. Finally, Maiga et al. [54] found a 75% reduction (95% CI [15%-94%]) in clinical malaria in children under five years of age after the CPSMC was implemented. However, they observed a decrease in malaria prevalence after SMC implementation, from 11.8% before the campaign to 6.1% after. Taken together, the results of these seven studies demonstrate the effectiveness of SMC in reducing malaria incidence in children under five in Sahelian zones and in areas where malaria is endemic. A second evaluation of four clinical trials [39,55–57] revealed a pooled effect size of 1.28 (95% CI [1.10, 1.46]) and a z-test statistic of 13.90 (p < 0.08). In the random-effects model, the effect size was lower but remained significant in terms of reducing malaria morbidity and mortality in children under five. Moderate heterogeneity was observed among these studies, with an I² of 56.2%, though no significant difference was detected by the Q test. Cairns et al. [56] found that SMC had a protective efficacy of 87.4% (95% CI [79.6%-92.2%]) against hospitalization or death due to malaria. SMC also showed a protective efficacy of 78.3% (95% CI [76.8-79.6]) against uncomplicated malaria. Konate et al. [39] reported that SMC led to significant reductions in clinical malaria incidence: 73% (HR = 0.27, 95% CI [0.18, 0.40]) in 2015 and 77% (HR = 0.23, 95% CI [0.15, 0.35]), in 2016 compared to October 2013. Aboubacar et al. [57] reported that SMC resulted in a 41% reduction in incidence and a 15% reduction in mortality compared to the 2011 incidence rate. In summary, these studies demonstrate the effectiveness of seasonal malaria chemoprevention in reducing malaria incidence in children in the studied regions, with reduction rates reaching 87.4% (Table 6).

**Table 6:** Quality Assessment risk in studies (RoB 2).

Authors (N°ref)	Publication journal	Biais in the randomization process	Biais related to deviations from planned interventions	Measurement bias	Missing data bias	Bias in the selection of reported results
Cairns (41)	Low	Low	Low	Low	Low	Low

Despite differences in study design (e.g., sample size, follow-up duration, location, climate, and sociocultural behavior), the two groups’ studies support the efficacy of SMC in reducing malaria incidence, prevalence, and mortality in children under five years of age in Sahelian zones. Published online data extensively documents the efficacy of seasonal malaria chemoprevention (SMC) in reducing the incidence and severity of malaria 28 days after the first dose. Analyses of randomized clinical trials and observational studies convincingly demonstrate that periodically administering antimalarial drugs during the high-risk season significantly reduces the incidence of malaria episodes and associated complications, particularly in children under five. For instance, a recent meta-analysis [50] reported a notable decrease in malaria incidence among children receiving SMC compared to those not receiving prophylaxis. Additionally, a prospective cohort study published in The Lancet [53] showed a substantial reduction in malaria-related mortality in children receiving a well-tolerated, regularly administered SMC regimen. These results support the efficacy of SMC as a public health strategy to reduce the malaria burden, complementing other malaria control interventions. However, continuous monitoring and adaptation to local contexts are necessary to maximize the benefits of SMC while minimizing potential risks, particularly in the context of emerging antimalarial drug resistance. Seasonal malaria chemoprevention (SMC) has attracted growing interest as a public health strategy to reduce the incidence and severity of malaria, particularly among high-risk populations, such as children in Sahelian endemic regions. Clinical trials and observational studies have shown that regularly administering antimalarial drugs during the high-risk season significantly reduces malaria episodes and associated complications, especially in children under five. This targeted prophylactic approach aims to suppress the parasite load and prevent symptomatic infections, thereby helping to reduce malaria-related morbidity and mortality. Additionally, SMC provides an opportunity to complement other malaria control interventions, such as intermittent preventive treatment for pregnant women, thereby strengthening the available tools to combat this disease. However, challenges remain regarding coverage, acceptability among children, drug resistance, and the sustainability of SMC programs. This underscores the need for an integrated and adaptive approach to maximize the long-term impact of SMC in reducing the malaria burden.

## Discussion

The majority of the studies included in this review reported the efficacy of seasonal malaria chemoprevention (SMC) in preventing and reducing the incidence of malaria in children under five years of age [33,39,51–60]. The results of a meta-analysis of evaluation studies corroborate the findings of individual studies and support the recent positive findings reported by the WHO. However, it is difficult to determine if the entire target population has been reached due to problems with the population census. Estimating SMC coverage rates remains a major challenge when assessing this strategy’s effectiveness. Overall coverage varies by region, often exceeding 100%. The main challenges to implementing SMC are: i) The logistical difficulty of reaching communities, particularly in areas where insecurity and accessibility issues are common; ii) The difficulty of tracking down the same child three or five times during the season to administer treatment; iii) The obstacles to establishing accurate censuses for effective logistical planning and realistic evaluation of effectiveness. A door-to-door strategy is often employed, costing several tens of millions of USD per campaign. This strategy mobilizes hundreds of full-time staff members throughout the transmission season, with some periods overlapping. However, some studies have reported cases of hospitalization among children who benefited from SMC [61]. There are many reasons for this, relating to the complexity of the implementation system. Often, only the first dose of SMC is administered under the supervision of healthcare workers. The second and third doses are given to parents/guardians for administration on subsequent days. Furthermore, the quantity of drugs provided to distributors is sometimes insufficient, making it impossible to repeat the treatment for children who vomit after the first dose (notably due to the bitter taste of Amodiaquine). The population generally accepted the strategy very well. A few adverse events [58] were reported, including vomiting, diarrhea, and skin rashes, which were mainly associated with amodiaquine. These events can occasionally compromise treatment acceptability. Therefore, it is crucial that Sahelian countries implementing SMC strengthen pharmacovigilance and report adverse events. This will also enable monitoring of the emergence of resistance and assessment of malaria infection incidence after the last SMC dose. One way to accomplish this follow-up is to assign a non-redundant number to each child during the SMC campaign to record all adverse effects and failures of protection at the local dispensary relevant to this number.

Finally, since SMC is always combined with other prevention strategies, such as the use of LLINs and IRS, it is difficult to attribute malaria control successes solely to SMC.

The cost and cumbersome nature of field campaigns make this strategy complex and entirely dependent on financial partners. No country can afford this strategy alone. Consequently, the system is not self-sustaining, which compromises its existence from the outset. To reduce costs, SMC campaigns are now combined with other programs, particularly malnutrition screening [17]. There are also plans to combine these efforts with malaria vaccination, which should benefit from a fairly similar booster schedule to that of SMC. Another difficulty is the failure to deliver the second and third doses at home [17]. Therefore, it is important for agents and/or community relays to return to households on days two and three to supervise home dosing. However, this is logistically and financially difficult to implement [62]. Similarly, seeing the same child three to five times during a transmission season remains challenging and compromises the efficacy of the treatment [63]. Finally, many children do not benefit from this strategy due to a lack of family registration [24]. Resistance to AQ and SP reduces the effectiveness of the strategy [64]. Studies published in recent years report an increase in resistance to sulfadoxine-pyrimethamine throughout Africa, with the prevalence of mutated plasmodial strains ranging from 15 to 20% [17,34,62]. Nevertheless, the combination of the two molecules maintains overall efficacy [65]. However, it would be preferable to reconsider this sulfadoxine-pyrimethamine-based strategy now in order to preserve these molecules, which are also used to prevent malaria in pregnant women [17]. To prevent the spread of resistance in Africa, particularly in Sahelian countries [42,66–68], the WHO recommends combining at least two drugs active against the parasite with long shelf lives [25,42,45,64]. The WHO also recommends regularly monitoring strain susceptibility [69] based primarily on searching for mutations that induce drug resistance in the parasite. The local population’s perception of the effectiveness of the strategy is a major factor in compliance. Therefore, failures should be analyzed to define alternative strategies. Conversely, in rural areas with low literacy rates, the local population may overestimate the efficacy of the strategy. It is difficult to convince mothers that a child who has received a dose of SMC medication can still be ill with malaria and require urgent medical attention. This can lead to accidents. Therefore, health education is a major factor in the effectiveness and safety of these campaigns.

Currently, SMC is only recommended for children under the age of five. However, in areas where malaria transmission is hypo- or mesoendemic, there has been a significant rise in cases among children over the age of five. These children are less well protected by current strategies. Depending on the local epidemiological context, it would make sense to extend SMC up to the age of 10 [70]. Each country should reanalyze epidemiological data relating to malaria in children aged 5–10 to detect rebounds in malaria-related morbidity and mortality and consider this extension. However, such an extension would considerably increase the cost of campaigns by requiring more treatments.

In the central and western Sahel, heavy rains have led to the formation of residual pools and floods that provide favorable breeding grounds for Anopheles larvae. Due to this excess rainfall, national epidemiological data, particularly in Niger [71], indicate an increase in malaria transmission in northern Sahel zones. New populations of children are at high risk of malaria and should be covered by the SMC. This strategy should be implemented in areas that receive less than 600 mm of rain per year in the Sahel-Saharan regions. Similarly, these residual bodies of water ensure the survival of larval breeding sites later in the year, leading to malaria transmission much later in the dry season, sometimes from December to March. National malaria control programs in these countries must consider treatment cycles during the dry season to protect children. Finally, changes in biotopes are leading to modifications in vector species mapping. Anopheles funestus [17] has rapidly returned to the Sahel, but most notably, *Anopheles Stephensis* [21,22,72] is progressively invading West and Central Africa. These changes in vectors will jeopardize malaria control strategies in Africa and the Sahel because forecasts predict that, by 2050, 60% of the population will live in urban areas [73].

Therefore, each region should adapt the start date and geographical scope of SMC locally according to climatic and ecosystem conditions rather than adhering to a national or sub-regional timetable, as is currently the case [74–77].

### Limits of this study

Ten years after the introduction of seasonal malaria chemoprevention (SMC) in Sahelian countries, this review assessed the effectiveness of SMC as a flagship malaria prevention strategy. The main limitation is the small number of high-quality evaluation studies available, undoubtedly due to the methodological and ethical challenges of conducting such studies. It is difficult to establish control groups that are not treated and that are subject to the same ecological conditions of transmission, and therefore malaria risk, as the groups treated with SMC. Creating treated and untreated zones would be politically and ethically challenging. Publication bias was explored using funnel plots. However, due to the small number of studies included (<10), their interpretation remains limited. The Egger’s test was not applied. An additional search of clinical trial registries was carried out to identify any unpublished studies.

## Conclusion

Seasonal malaria chemoprevention (SMC) has been shown to significantly reduce malaria-related morbidity and mortality in children under five in Sahelian regions, as evidenced by the observational studies and clinical trials included in this systematic review and meta-analysis. The pooled analysis revealed substantial reductions in the incidence of uncomplicated malaria (up to 75.2%), severe malaria (26%), and malaria mortality (48%), confirming SMC’s effectiveness in areas of seasonal transmission.

However, several challenges stand in the way of its optimal implementation and sustainability. These challenges include logistical constraints, such as access to remote and unstable areas; incomplete adherence to treatment, particularly for the second and third doses; and inaccurate estimates of target populations, which compromise administrative coverage in certain localities. Additionally, the emergence of resistance to amodiaquine and sulfadoxine-pyrimethamine (AQSP) threatens the future efficacy of SMC, necessitating continuous molecular monitoring and possible modifications to the treatment regimen.

The heavy dependence of National Malaria Control Programs (NMCPs) on external funding raises concerns about sustainability, and operational complexities, including the need for repeated monthly organization, put a heavy burden on health systems. Future adaptations should consider extending SMC eligibility to older children (ages 5–10) in areas where malaria transmission is seasonal. The campaign schedule should be adjusted according to changes in transmission due to climate. SMC should be integrated with complementary interventions, such as malaria vaccination (RTS, S/AS01, R21/Matrix-M) and vector control, to achieve a synergistic impact.

This study provides solid evidence confirming the remarkable efficacy of seasonal malaria chemoprevention (SMC) as a major public health intervention in Sahelian regions. Consolidated data demonstrate an impressive reduction in cases of simple and severe malaria and malaria-related deaths in children under five, thus validating current WHO recommendations.

Currently, SMC is an essential component of the fight against seasonal malaria; however, its future depends on the ability of National Malaria Control Programs (NMCPs) to adapt to emerging challenges.

This systematic review assesses the impact of SMC after ten years of deployment in Sahelian countries. The review aims to determine if SMC has significantly reduced the incidence and prevalence of malaria in children under five. Finally, it identifies persistent challenges and opportunities for program optimization.

## Supporting information

S1 Table Description of the studies selected for analysis. (PDF)

S2 Table Summaries and results of the studies included in meta-analysis. (PDF)

S1 Checklist PRISMA Checklist. (PDF)
