# Supplementary material for: Status and prospects of seasonal malaria chemoprevention among children in Sahelian countries: A systematic review and meta-analysis
Source: PLOS Glob Public Health. 2025 Sep 12;5(9):e0005124. doi: 10.1371/journal.pgph.0005124 (PMC12431219; doi:10.1371/journal.pgph.0005124)
Supplement: S1 Table — (PDF) [file pgph.0005124.s001.pdf]

Supplementary Table I: Studies selected

Journal	ID	Authors	Year of publication	Abstract titles	Final decision	Reasons
Trials [Electronic Resource] 23(1): 627.	1	Afolabi	2022	Safety and effectiveness of delivering mass drug administration for helminths through the seasonal malaria chemoprevention platform among Senegalese children: study protocol for a randomised controlled trial.	Exclude	excluded because it is a study protocol, and does not contain SMC results
Journal of Infectious Diseases 221(1): 138-145.	2	Attaher	2020	Effect of Seasonal Malaria Chemoprevention on Immune Markers of Exhaustion and Regulation.	includes	study meeting our study protocol, inclusion criteria and homogeneous enough for meta-analysis.
The Lancet 396(10265): 1829-1840.	3	Baba	2020	Effectiveness of seasonal malaria chemoprevention at scale in west and central Africa: an observational study.	includes	study meeting our study protocol, inclusion criteria and homogeneous enough for meta-analysis.
American Journal of Tropical Medicine and Hygiene 103(5 SUPPL): 407-408.	4	Baker	2020	Extending seasonal malaria chemoprevention in burkina faso to five cycles to coincide with the start of the rainy season in the cascades region: Results from a pilot study to assess feasibility, acceptability, cost and impact on malaria incidence.	Exclude	Evaluates feasibility, acceptability, cost and impact on the incidence of malaria. may not provide results on the effectiveness of SMC
American Journal of Tropical Medicine and Hygiene 95(5 Supplement 1): 6-7.	5	Barry	2016	Comparison of three versus four rounds of seasonal malaria chemoprevention on the incidence of clinical malaria in mali.	Exclude	It was difficult to extract the effectiveness of SPC alone in this study, as it was combined with other strategies such as the use of long-lasting impregnated nets
American Journal of Tropical Medicine and Hygiene 99(4 Supplement): 663.	6	Bonkoungou	2018	Seasonal malaria chemoprevention, an effective intervention for reducing malaria morbidity and mortality.	Exclude	Summary with less information on the results of the SMC study
Antimicrobial Agents and Chemotherapy 54(3): 1265-1274.	7	Cairns	2010	Amodiaquine dosage and tolerability for intermittent preventive treatment to prevent malaria in children.	Exclude	the results of this study discuss the dosage and tolerability of amodiaquine as part of intermittent preventive treatment to prevent malaria in children. however, we are studying the efficacy of combined AQSP in children under 5 years of age benefiting from the strategy
Nature Communications 3.	8	Cairns	2012	Estimating the potential public health impact of seasonal malaria chemoprevention in African children.	Exclude	this study evaluates the seasonality of malaria incidence data while defining a seasonality indicator based on rainfall. not the efficacy results of a SMC intervention.
PLoS Medicine / Public Library of Science 17(8): e1003214.	9	Cairns	2020	Evaluation of seasonal malaria chemoprevention in two areas of intense seasonal malaria transmission: Secondary analysis of a household-randomised, placebo-controlled trial in Hounde District, Burkina Faso and Bougouni District, Mali.	includes	study meeting our study protocol, inclusion criteria and homogeneous enough for meta-analysis.
PLoS Medicine / Public Library of Science 13(11): e1002175.	10	Cisse	2016	Effectiveness of Seasonal Malaria Chemoprevention in Children under Ten Years of Age in Senegal: A Stepped-Wedge Cluster-Randomised Trial.	includes	study meeting our study protocol, inclusion criteria and homogeneous enough for meta-analysis.
Frontiers in Neurology 13 (no pagination)(988960).	11	Coulibaly	2022	Shifts in the clinical epidemiology of severe malaria after scaling up control strategies in Mali.	Exclude	The study describes the clinical phenotypes of severe forms encountered by participants enrolled in Bandiagara, Bamako and Sikasso, Mali based on retrospective data of severe malaria.
Antimicrobial Agents and Chemotherapy 62(5).	12	de Kock	2018	Population Pharmacokinetic Properties of Sulfadoxine and Pyrimethamine: a Pooled Analysis To Inform Optimal Dosing in African Children with Uncomplicated Malaria.	Exclude	this study reports more on the nutritional status of children exposed to sulfadoxine and pyrimethamine, but no results on SMC.
Malaria Journal 20(1): 274.	13	de Wit	2021	Nutritional status in young children prior to the malaria transmission season in Burkina Faso and Mali, and its impact on the incidence of clinical malaria.	Exclude	This study was excluded because it reports the incidence of malaria in malnourished children and does not include results on the efficacy of SMC itself.

Annals of Nutrition and Metabolism 71(Supplement 2): 370-371.	14	Diarra	2017	Impact of micronutrient powders combined with malaria chemoprevention on anemia, malaria and cognitive development: A cluster-randomized study in malian children.	Exclude	the results of the study are less informative about the efficacy of SMC in reducing malaria in children under 5. this study focuses on anemia and stunting due to malaria.
Malaria Journal 16(1): 325.	15	Diawara	2017	Measuring the impact of seasonal malaria chemoprevention as part of routine malaria control in Kita, Mali.	includes	study meeting our study protocol, inclusion criteria and homogeneous enough for meta-analysis.
Malaria Journal 20(1): 128.	16	Diawara	2021	Cost-effectiveness of district-wide seasonal malaria chemoprevention when implemented through routine malaria control programme in Kita, Mali using fixed point distribution.	Exclude	This study was excluded because its results focus exclusively on the costs of implementing SMC
American Journal of Tropical Medicine and Hygiene 101(5 Supplement): 314.	17	Diawara	2019	Impact of seasonal malaria chemoprevention among children five to ten years of age in kita and bafoulabe districts, mali.	includes	study meeting our study protocol, inclusion criteria and homogeneous enough for meta-analysis.
Malar J. 2008 Jul 8:7:123.	18	Dicko	2008	Impact of intermittent preventive treatment with sulphadoxine-pyrimethamine targeting the transmission season on the incidence of clinical malaria in children in Mali.	Exclude	this study was excluded because it only included results for sulphadoxine-pyrimethamine, and not for amodiquine, whereas according to WHO recommendations, SMC is a combination of the two in endemic countries
Clinical Pharmacology and Therapeutics 107(5): 1179-1188.	19	Ding	2019	Adherence and population pharmacokinetic properties of amodiaquine when used for seasonal malaria chemoprevention in African children.	Exclude	this study was excluded because it looked instead at the concentration profiles of amodiaquine and desethylamodiaquine, which were modeled with two- and three-compartment regimens, respectively. also, it showed that less than 20% of children took all doses of SMC, highlighting an urgent need to improve adherence to treatment. so the results are less informative
Bulletin De L Academie Nationale De Medecine 200(3): 453-466.	20	Doumbo	2016	Malaria is still a leading cause of fever and death among children and pregnant women in Africa in 2015.	Exclude	the study was excluded because it reported the results of a new SMC Plus strategy (AQ-SP+Azithromycin), and information on the efficacy of SMC in reducing malaria was less informative;also age is off-target.
PLoS Un. 10.1371/journal.pone.0210692	21	Abigail Ward	2019	Chimioprévention du paludisme saisonnier associée à la prévention de la malnutrition dans le nord du Nigéria : un essai pragmatique (étude SMAMP) avec cas-témoins imbriqués	Exclude	excluded because the results are focused on malnutrition, and the results are less informative.
Tropical Medicine & International Health 22: 59-60.	22	Druetz	2017	Seasonal malaria chemoprevention in Burkina Faso protects children against malaria and anaemia under routine program implementation.	Exclude	although the study reports some results on the reduction in malaria prevalence after MCH and some results on parasitaemia and overall coverage, the data were not detailed enough to be homogeneous with other studies for a meta-analysis.
American Journal of Tropical Medicine & Hygiene 98(2): 524-533.	23	Druetz	2018	Impact Evaluation of Seasonal Malaria Chemoprevention under Routine Program Implementation: A Quasi-Experimental Study in Burkina Faso.	Exclude	this study was excluded because the results of this study on MCH showed a reduction in the punctual and periodic prevalence of parasitaemia and the probability of having moderate or severe anaemia, as well as the coverage of children in the first cycle. not enough information on the impact of MCH in reducing the incidence of malaria after implementation of the intervention.
Cochrane Database of Systematic Reviews 7: 7.	24	Esu	2021	Intermittent preventive treatment for malaria in infants.	Exclude	Although the results of this study are well presented, it reports only results on IPTi with SP in reducing episodes of clinical malaria, anemia, parasitemia and hospital admissions. in our systematic review we evaluate the combination of AQSP together in reducing episodes.

The Lancet Global Health 9(2): e199-e208.	25	Gilmartin	2021	Seasonal malaria chemoprevention in the Sahel subregion of Africa: a cost-effectiveness and cost-savings analysis.	Exclude	The study was excluded because the results evaluated the total cost of CMS for the seven African countries.
Malaria Journal 16.	26	Greenwood	2017	Seasonal vaccination against malaria: a potential use for an imperfect malaria vaccine.	Exclude	This study was excluded because its results show that SMC does not offer complete protection and is difficult to administer.and reports the risk of resistance to the drugs, sulfadoxine-pyrimethamine and amodiaquine, currently used for SMC. And talks about the malaria vaccine, RTS,S/AS01.
Journal of Public Health and Epidemiology 14(4): 166-172.	27	Issaka	2022	Seasonal malaria chemoprevention coverage in Burkina Faso in 2018: descriptive analysis of mothers' knowledge and attitude during cross sectional survey.	Exclude	This study was excluded because it reported the results of an assessment of the mother's knowledge of MCH and also the effects of MCH on the health of their children.
Malaria Journal 19(1): 103.	28	Issiaka	2020	Impact of seasonal malaria chemoprevention on hospital admissions and mortality in children under 5 years of age in Ouelessebougou, Mali.	includes	study meeting our study protocol, inclusion criteria and homogeneous enough for meta-analysis.
PLoS ONE [Electronic Resource] 11(10): e0162563.	29	Ndiaye JL	2016	Safety of Seasonal Malaria Chemoprevention (SMC) with Sulfadoxine-Pyrimethamine plus Amodiaquine when Delivered to Children under 10 Years of Age by District Health Services in Senegal: Results from a Stepped-Wedge Cluster Randomized Trial.	Exclude	this study was excluded because it reports the results of the surveillance system in place to record all deaths and all cases of malaria diagnosed by pharmacovigilance to detect adverse drug reactions and coverage after three years of intervention.
American Journal of Tropical Medicine and Hygiene 105(5): 364-364.	30	Kamate	2021	PRELIMINARY FINDINGS ON IMPACT OF SEASONAL MALARIA CHEMOPREVENTION ON MALARIA INCIDENCE AMONG CHILDREN 5 TO 10 YEARS OF AGE IN A HIGH MALARIA PREVALENCE REGION IN MALI (SIKASSO).	Exclude	this study was excluded because its summary was not totally accessible
Malaria Journal 21(1): 143.	31	Kirakoya-Samadoulougou	2022	Assessing the effect of seasonal malaria chemoprevention on malaria burden among children under 5 years in Burkina Faso.	Exclude	This study was excluded firstly because we do not have information on the two SMC series and cannot at this stage say whether they are comparable to the interventions studied. The data on incidence rates before and after the intervention or in a control group are not clear enough, yet they provide crucial data such as (IRR, IC 95%, p-value)
Mali Sante Publique [Internet]. 4 août 2021 [cité 29 nov. 2024];11(1):85-93.	32	Diarra NH	2021	Amélioration de la qualité de la mise en œuvre de la chimio prévention du paludisme saisonnier : Recherche formative à Koulikoro au Mali en 2020	Exclude	This study was excluded; although it addresses important qualitative elements (perception, barriers, and acceptability),<br>Although the study addresses important qualitative elements, it lacks quantitative data directly measuring the impact of MCH on the reduction of malaria cases and the effects on morbidity and mortality.
Malaria Journal 19(1): 137.	33	Konate	2020	Effect of routine seasonal malaria chemoprevention on malaria trends in children under 5 years in Dangassa, Mali.	includes	study meeting our study protocol, inclusion criteria and homogeneous enough for meta-analysis.
Malaria Journal 20(1): 1.	34	Mahaman Moustapha	2021	Evidence that seasonal malaria chemoprevention with SPAQ influences blood and pre-erythrocytic stage antibody responses of Plasmodium falciparum infections in Niger.	Exclude	This study was excluded because it did not provide clear data on standardized indicators such as malaria incidence or associated hospitalization. No explicit comparisons were made to calculate RR, OR, HR.
Malaria Journal 16(1): 289.	35	Mahamar	2017	Effect of seasonal malaria chemoprevention on the acquisition of antibodies to Plasmodium falciparum antigens in Ouelessebougou, Mali.	Exclude	This study was excluded because SMC was used to compare the reduction in seropositivity to MSP-142 in two

						groups, and does not provide quantitative results on the efficacy of SMC itself.
⟨hal-04480426⟩	36	Daniel Christian Koko	2021	Analyse des attitudes et des pratiques influençant l'observance au traitement de la Chimio-prévention du Paludisme Saisonnier chez les enfants de moins de 5 ans de la région de Dosso - Niger	Exclude	This study was excluded because it reports on the attitudes and practices of community agents and parents regarding medication compliance during the CPS campaign.
Malaria Journal 21(1): 39.	37	Mahamar	2022	Effect of three years' seasonal malaria chemoprevention on molecular markers of resistance of Plasmodium falciparum to sulfadoxine-pyrimethamine and amodiaquine in Ouelessebougou, Mali.	Exclude	This study was excluded because it reports the results of a study carried out on 100 samples analyzed by PCR 1,164 samples analyzed by sequencing to highlight molecular markers of resistance. it does not report quantitative results on SPC.
International Journal of Environmental Research & Public Health [Electronic Resource] 17(18): 11.	38	Maiga	2020	Two-Year Scale-Up of Seasonal Malaria Chemoprevention Reduced Malaria Morbidity among Children in the Health District of Koutiala, Mali.	includes	study meeting our study protocol, inclusion criteria and homogeneous enough for meta-analysis.
Africa health (Online) 37(1): 22-23.	39	Misiorowska	2014	Protecting the Sahel's Children with Seasonal Malaria Chemoprevention.	Exclude	Study excluded because abstract gives less quantitative information on SMC.
Plos One 11(12).	40	Ndiaye	2016	Safety of Seasonal Malaria Chemoprevention (SMC) with Sulfadoxine-Pyrimethamine plus Amodiaquine when Delivered to Children under 10 Years of Age by District Health Services in Senegal: Results from a Stepped-Wedge Cluster Randomized Trial (vol 11, e0162563, 2016).	Exclude	study excluded as it reports data on the implementation oSMC in African countries and the tolerability of the Our results show that SMC is well tolerated in children under 5 years of age and in older children. However, pharmacovigilance must be maintained where SMC is implemented, and the strengthening of national pharmacovigilance systems must be included in SMC implementation plans.
Pharmaceutical Medicine 32(3): 189-200.	41	Ndiaye	2018	Evaluation of Two Strategies for Community-Based Safety Monitoring during Seasonal Malaria Chemoprevention Campaigns in Senegal, Compared with the National Spontaneous Reporting System.	Exclude	This study was excluded because its results mainly focus on the adverse effects following the distribution of AQSP-based CPS to children under 5 years old. It does not highlight the quantitative results of SMC.
American Journal of Tropical Medicine and Hygiene 97(5 Supplement 1): 134.	42	Ndiaye	2017	Impact of seasonal malaria chemoprevention after 3 years at scale in Southern Senegal.	Exclude	Study excluded, as the summary is less informative, and does not include all quantitative information.
Malaria Journal 7.	43	Ndugwa	2008	Comparison of all-cause and malaria-specific mortality from two West African countries with different malaria transmission patterns.	Exclude	Although the study reports results on infant mortality in two countries between 1960 and 2004, the abstract study was excluded because the results do not provide statistics on the effectiveness of SMC in reducing the incidence of malaria.
Nuclear Physics B, Volume 433, Issue 1, 1995,	44	S. Narison	1995	Target independence of the EMC-SMC effect	Exclude	Although the study presents interesting statistics, the abstract does not provide results on the intervention of SPC in reducing the incidence of malaria, which is why the study is excluded.
Infection and Drug Resistance, 15, 4517–4527.	45	Séni Nikiema	2022	Seasonal Malaria Chemoprevention Implementation: Effect on Malaria Incidence and Immunity in a Context of Expansion of P. falciparum Resistant Genotypes with Potential Reduction of the Effectiveness in Sub-Saharan Africa	Exclude	The present study examines the impact of SMC on malaria morbidity, parasite resistance to antimalarial drugs, molecular immunity and immunity affecting malaria incidence in children. But it does not provide qualitative data on SMC.

American Journal of Tropical Medicine and Hygiene 77(3): 411-417.	46	Ntab	2007	Impact of intermittent preventive anti-malarial treatment on the growth and nutritional status of preschool children in rural Senegal (West Africa).	Exclude	This study reports the impact of a randomized, double-blind, placebo-controlled trial of seasonal intermittent preventive antimalarial treatment on the growth and nutritional status of 1,063 Senegalese preschool children. A combination of artesunate and sulfadoxine-pyrimethamine was administered monthly from september to november. but did not report essential quantitative results on CPS and also AQSP. that's why it's excluded.
American Journal of Tropical Medicine and Hygiene 101(5 Supplement): 302.	47	Obiero	2019	The effect of adding azithromycin to the antimalarials (sulphadoxine/pyrimethamine and amodiaquine) used for seasonal malaria chemoprevention on the immune response to plasmodium falciparum.	Exclude	study excluded because its abstract is less informative about the main results.
American Journal of Tropical Medicine and Hygiene 95(5 Supplement 1): 267.	48	Ouedraogo	2016	Effective scaling-up of seasonal malaria chemoprevention in burkina faso.	Exclude	study excluded because its abstract is less informative about the main results.
Ghana Medical Journal 56(2): 64-70.	49	R Adjei	2022	Effectiveness of seasonal malaria chemoprevention in reducing under-five malaria morbidity and mortality in the Savannah Region, Ghana.	Exclude	the study reports key results on the reduction of malaria-related morbidity and mortality, with well-argued statistics of 17% ($p<0.01$) and 67% ($p=0.047$). but the main results are missing, especially the quantitative ones on SMC.
J Trop Med . 2020 Jul 1:2020:9372457	50	J P Ampe	2020	Impacts of Seasonal Malaria Chemoprevention on Malaria Burden among under Five-Year-Old Children in Borno State, Nigeria	Exclude	this well-structured study reports results on the prevalence of malaria and anemia with good statistics, but does not report data on OR, RR, HR. this is why it was excluded.
International Journal of Environmental Research & Public Health [Electronic Resource] 18(2): 19.	51	Sacko	2021	Evolution of Malaria Incidence in Five Health Districts, in the Context of the Scaling Up of Seasonal Malaria Chemoprevention, 2016 to 2018, in Mali.	Exclude	Although the study reports excellent results on the reduction of overall incidence in all the districts studied in relation to components such as relative humidity, rainfall and minimum temperature in children under 5 years of age, it does not report quantitative results on the efficacy of SMC, which is why it was excluded.
American Journal of Tropical Medicine and Hygiene 97(5 Supplement 1): 531.	52	Sagara	2017	Seasonal malaria chemoprevention scaling up and its impact assessment in Mali.	Exclude	results in the summary, less informative, little quantitative data on SMC
International Journal of Biological and Chemical Sciences 11(2): 685-693.	53	Salissou	2017	Estimation of public health impact of seasonal malaria chemoprevention in Niger. [French].	Exclude	SMC reduced the incidence of uncomplicated malaria by 37%, severe malaria by 26.5%, convulsions by 19.9%, comas by 13.2%, hospitalizations by 23%, and mortality by 48.3%, with no impact on anemia. it did not show OR,RR and other results
International Journal of Biological and Chemical Sciences 10(6): 2710-2715.	54	Salissou	2016	Perception of the seasonal malaria chemoprevention in Niger. [French].	Exclude	This study reports on the satisfaction of parents of children receiving CPS, SMC coverage and the main adverse events reported. It does not report the essential outcomes of CPS. This is why it was excluded.
American Journal of Tropical Medicine and Hygiene 101(5 Supplement): 273.	55	Sangare	2019	Seasonal malaria chemoprevention and compliance during four monthly treatments with sulfadoxine-pyrimethamine and amodiaquine at 3 study sites in Mali.	Exclude	study excluded because study summary is not accessible
Tropical Medicine & International Health 20: 78-79.	56	Saye	2015	Malaria chemoprevention, undernutrition and anaemia in children: findings from three randomized intervention trials in Southern Mali.	Exclude	study excluded because study summary is not accessible

Bulletin de la Societe de Pathologie Exotique 55(2): 280-290.	57	Schneider	1962	Malaria Chemoprophylaxis by means of Weekly Distributions of Chloroquine or of combined Chloroquine-Primaquine-Pyrimethamine. [French].	Exclude	the study was excluded because it reports the results of a study on the association between chloroquine-primaquine-pyrimethamine and insufficient information on SMC.
Malaria Journal 19(1): 238.	58	Some	2020	Investigating selected host and parasite factors potentially impacting upon seasonal malaria chemoprevention in Bama, Burkina Faso.	Exclude	The study was excluded because, although well structured, it reported PCR results shortly after SMC administration in children under 5 years of age.
Archives of Public Health 80(41).	59	Sondo	2022	Boosting the impact of seasonal malaria chemoprevention (SMC) through simultaneous screening and treatment of household members of children receiving SMC in Burkina Faso: a protocol for a randomized open label trial.	Exclude	study excluded as it is still a study protocol - no results at this stage.
Trials [Electronic Resource] 22(1): 360.	60	Sondo	2021	Assessment of a combined strategy of seasonal malaria chemoprevention and supplementation with vitamin A, zinc and Plumpy'Doz TM to prevent malaria and malnutrition in children under 5 years old in Burkina Faso: a randomized open-label trial (SMC-NUT).	Exclude	very interesting study, but excluded because it reports more results on children's nutritional status.
Malaria Journal 8(1) (no pagination)(257).	61	Souares	2009	Self-reported data: A major tool to assess compliance with anti-malarial combination therapy among children in Senegal.	Exclude	the study was excluded because it reports more results on self-reported data in children benefiting from SMC not enough information on SMC.
American Journal of Tropical Medicine and Hygiene 95(5): 115-115.	62	Sylla	2017	IMMUNOLOGICAL EFFECT OF SEASONAL MALARIA CHEMOPREVENTION (SMC) WITH SULFADOXINE-PYRIMETHAMINE (SP) AND AMODIAQUINE (AQ) IN CHILDREN UNDER 10 YEARS IN THE SOUTHEASTERN PART OF SENEGAL.	Exclude	this study was also excluded because its results focused on the prevalence of malaria and those of the seroprevalence of anti-MSP1_42 anti-AMA1 antibodies, not enough information on SMC.
Parasite Epidemiology and Control 3(2): 96-105.	63	Thera	2018	School-aged children based Seasonal Malaria Chemoprevention using artesunate-amodiaquine in Mali.	Exclude	Although this study reported a protective efficacy of 85% against clinical malaria, the results were not complete enough for a meta-analysis.
Transactions of the Royal Society of Tropical Medicine & Hygiene 108(1): 13-21.	64	Tine	2014	Feasibility, safety and effectiveness of combining home based malaria management and seasonal malaria chemoprevention in children less than 10 years in Senegal: a cluster-randomised trial.	Exclude	This study was excluded because its results were essentially based on the adjusted rate ratio for the incidence of malaria attacks in intervention and control communities, and the results were not complete enough for a meta-analysis.
Malaria Journal 21(1): 65.	65	Toure	2022	Trends in malaria epidemiological factors following the implementation of current control strategies in Dangassa, Mali.	Exclude	This study was excluded because its results are less informative about SPC, and speak instead of the prevalence of malaria infection and bite rates.
Malaria Journal 20(1).	66	Yameogo	2021	Effect of seasonal malaria chemoprevention plus azithromycin on Plasmodium falciparum transmission: gametocyte infectivity and mosquito fitness.	Exclude	This well-structured study was excluded because on the one hand it's difficult to determine the efficacy of SMC, and on the other, its results speak more about gametocytes and oocysts.
Scientific Reports 12(1): 1402.	67	Yaro	2022	Risk of Plasmodium falciparum infection in south-west Burkina Faso: potential impact of expanding eligibility for seasonal malaria chemoprevention.	Exclude	This study was excluded because information on the efficacy of SMC is less consistent.
Antimicrobial Agents & Chemotherapy 59(8): 4387-4396.	68	Zongo	2015	Randomized Noninferiority Trial of Dihydroartemisinin-Piperaquine Compared with Sulfadoxine-Pyrimethamine plus Amodiaquine for Seasonal Malaria Chemoprevention in Burkina Faso.	Exclude	This study was excluded because it reports the risk of malaria attacks, and also pfdhfr and pfdhps mutations, in children who received SPAQ

American Journal of Tropical Medicine and Hygiene 97(5 Supplement 1): 134.	69	Manga, et al	2022	Malaria parasite carriage before and two years after the implementation of seasonal malaria chemoprevention: a case study of the Saraya health district, southern Senegal	includes	study meeting our study protocol, inclusion criteria and homogeneous enough for meta-analysis.
Health Science and disease	70	Aboubacar et al.	2021	Impact de la Chimio Prévention du Paludisme sur la Morbidité et la Mortalité des Enfants de 3-59 Mois dans le district Sanitaire de Diré Mali	includes	study meeting our study protocol, inclusion criteria and homogeneous enough for meta-analysis.
American Journal of Tropical Medicine and Hygiene 101(5 Supplement 1): 519.	71	Angoran-Benie	2019	Community-based to reach malnourished infants from 6 months to 5 years during a seasonal malaria chemoprevention (SMC) campaign in remote area in Niger	Exclude	less accessible and less instructive summary
