# Supplementary material for: Status and prospects of seasonal malaria chemoprevention among children in Sahelian countries: A systematic review and meta-analysis
Source: PLOS Glob Public Health. 2025 Sep 12;5(9):e0005124. doi: 10.1371/journal.pgph.0005124 (PMC12431219; doi:10.1371/journal.pgph.0005124)
Supplement: S2 Table — (PDF) [file pgph.0005124.s002.pdf]

Supplementary Table II Summary for each study

Study title	authors	Years of publication	Results/ summary
Effectiveness of seasonal malaria chemoprevention at scale in west and central Africa: an observational study	Baba, et al.	2020	In 779 individual case safety reports over 2015–16, 36 serious adverse drug reactions were reported (one child with rash, two with fever, 31 with gastrointestinal disorders, one with extrapyramidal syndrome, and one with Quincke's oedema). No cases of severe skin were reported. SMC treatment was associated with a protective effectiveness of 88·2% (95% CI 78·7–93·4) over 28 days in case-control studies (2185 cases of confirmed malaria and 4370 controls). In Burkina Faso and The Gambia, implementation of SMC was associated with reductions in the number of malaria deaths in hospital during the high transmission period, of 42·4% (95% CI 5·9 to 64·7) in Burkina Faso and 56·6% (28·9 to 73·5) in The Gambia. Over 2015–16, the estimated reduction in confirmed malaria cases at outpatient clinics during the high transmission period in the seven countries ranged from 25·5% (95% CI 6·1 to 40·9) in Nigeria to 55·2% (42·0 to 65·3) in The Gambia
Effectiveness of Seasonal Malaria Chemoprevention in Children under Ten Years of Age in Senegal: A Stepped-Wedge Cluster-Randomised Trial	Cissé, et al.	2016	About 780,000 treatments were administered over three years. Coverage exceeded 80% each month. Mortality, the primary endpoint, was similar in SMC and control areas (4.6 and 4.5 per 1000 respectively in children under 5 years and 1.3 and 1.2 per 1000 in children 5-9 years of age; the overall mortality rate ratio [SMC: no SMC] was 0.90, 95% CI 0.68-1.2, p = 0.496). A reduction of 60% (95% CI 54%-64%, p < 0.001) in the incidence of malaria cases confirmed by a rapid diagnostic test (RDT) and a reduction of 69% (95% CI 65%-72%, p < 0.001) groups was reduced by 29% (95% CI 21%-35%, p < 0.001). One hundred and twenty-three children were admitted to hospital with a diagnosis of severe malaria, with 64 in control areas and 59 in SMC areas, showing a reduction in the incidence rate of severe disease of 45% (95% CI 5%-68%, p = 0.031). Estimates of the reduction in the
Measuring the impact of seasonal malaria chemoprevention as part of routine malaria control in Kita, Mali	Diawara, et al	2017	Prevalence of parasitaemia was similar in intervention and comparison districts prior to SMC (23.4 vs 29.5%, p = 0.34) as was the prevalence of malaria illness (2.4 vs 1.9%, p = 0.75). After SMC, parasitaemia prevalence fell to 18% in the intervention district and increased to 46% in the comparison district [difference-in-differences (DD) OR = 0.35; 95% CI 0.20–0.60]. Prevalence of malaria illness fell to a greater degree in the intervention district versus the comparison district (DD OR = 0.20; 95% CI 0.04–0.94) and the same for moderate anaemia (Hb < 8 g/dL) (DD OR = 0.26, 95% CI 0.11–0.65). The frequency of the quintuple mutation (dhfr N51I, C59R and S108N + dhps A437G and K540E) remained low (5%) before and after intervention in both districts.

Effect of seasonal malaria chemoprevention in children between 5 and 9 years old in Kita and Bafoulabe districts, Mali	Diawara, et al	2017	in July 2017, 633 children aged 60–120 months old were enrolled at the Kita and Bafoulabe study sites (n = 310 and n = 323, respectively). Parasitemia prevalence was similar in the intervention and comparison districts prior the SMC campaign (27.7% versus 21.7%, p = 0.07). Mild anemia was observed in 14.2% children in Kita and in 10.5% of children in Bafoulabé. At the Kita site, household surveys showed an SMC coverage rate of 89.1% with a response rate of 93.3% among child caregivers. The most common adverse event reported by parents was drowsiness (11.8%). One year following SMC implementation in the older age group in Kita, the coverage of three doses per round was 81.2%. Between the baseline and endline surveys, there was a reduction in parasitemia prevalence of 40% (OR = 0.60, CI: 0.41–0.89).
Impact of seasonal malaria chemoprevention on hospital admissions and mortality in children under 5 years of age in Ouelessebougou, Mali	Issiaka, et al	2020	A total of 6638 children under 5 years of age were surveyed, 2759 children in the SMC intervention areas and 3879 children in the control areas. All causes mortality rate per 1000 person-years was 8.29 in the control areas compared to 3.63 in the intervention areas; age and gender adjusted mortality rate ratio 0.44 (95% CI 0.22–0.91), p = 0.027. The incidence rate of all causes hospital admissions was 19.60 per 1000 person-years in the intervention group compared to 33.45 per 1000 person-years in the control group, giving an incidence rate ratio (IRR) adjusted for age and gender of 0.61 (95% CI 0.44–0.84), p = 0.003.
Two-Year Scale-Up of Seasonal Malaria Chemoprevention Reduced Malaria Morbidity among Children in the Health District of Koutiala, Mali	Maiga, et al	2020	Results: Overall, 662 children were included in 2012, and 670 in 2014. Children in 2014 versus those surveyed in 2012 showed reduced proportions of malaria infection (12.4% in 2014 versus 28.7% in 2012 (p = 0.001)), clinical malaria (0.3% versus 4.2%, respectively (p < 0.001)), and anemia (50.1% versus 67.4%, respectively (p = 0.001)). A propensity score approach that accounts for environmental differences showed that SMC conveyed a significant protective effect against malaria infection (IR = 0.01, 95% CI (0.0001; 0.09), clinical malaria (OR = 0.25, 95% CI (0.06; 0.85))
Malaria parasite carriage before and two years after the implementation of seasonal malaria chemoprevention: a case study of the Saraya health district, southern Senegal	Manga, et al	2017	A total of 2008 children were included with a mean average age of 4.81 (+/-2.73) years. Of the study population, 50.33% were more than five years old and 50.3% were male. In 2013, mosquito net ownership was 99.4 % before the SMC campaign and 97.4% after. In 2015, it was 36.6% before and 45.8% after the campaign. In 2013, the prevalence of plasmodium carriage was 11.8% before and 6.1% after the SMC campaign. In 2015, the prevalence was 4.9% before the administration of SMC and this increased up to 15.3% after. Malaria prevalence was high among children over five years old and in boys.

3
